# An Unusual Histopathological Presentation of Mandibular Osteosarcoma

**DOI:** 10.1007/s12105-024-01724-4

**Published:** 2024-11-19

**Authors:** Saja A. Alramadhan, Rutvi Vyas, Donald M. Cohen, Indraneel Bhattacharyya, Mohammed N. Islam, John D. Reith

**Affiliations:** 1 Department of Oral and Maxillofacial Surgery and Pathology, University of Mississippi Medical Center School of Dentistry, 2500 N. State St, Jackson, MS 39213 USA; 2 Division of Clinical Dentistry, Detroit Mercy School of Dentistry, Detroit, MI USA; 3 Department of Oral and Maxillofacial Diagnostic Sciences, University of Florida College of Dentistry, Gainesville, FL USA; 4 Department of Pathology, Cleveland Clinic, Cleveland, OH USA

**Keywords:** Osteosarcoma, Jaw osteosarcoma, Gnathic osteosarcoma, Osteogenic sarcoma, Bone tumors, Glandular architectures

## Abstract

Jaw osteosarcoma (JOS) is a rare, distinct variant that differ from long bone osteosarcoma (LBOS) in several aspects. JOS typically appears about twenty years later than LBOS, displays a lower propensity for metastasis to other organs, and exhibits better survival rates. The dissimilarities in clinical and biological behavior between JOS and LBOS are likely due, at least in part, to variations in their respective microenvironments. In this report, we present a case of OS affecting the mandible in a young patient. This case displayed classic radiographic features but a unique histopathological presentation, posing a diagnostic challenge for pathologists, especially if encountered in small biopsies.

## Image Presentation

A 27-year-old female presented with a large, destructive mass on the right mandible, extending into the adjacent buccal gingival space. The mass caused significant pain and difficulty eating. The patient reported that the mass had been present and growing for several months, with recent noticeable external enlargement. The patient had a history of smoking (>10pack-years) but quit two months prior to presentation and never used smokeless tobacco. She denied any weight loss, had no significant past medical or surgical history, and was not taking any medications.

**Fig. 1:**
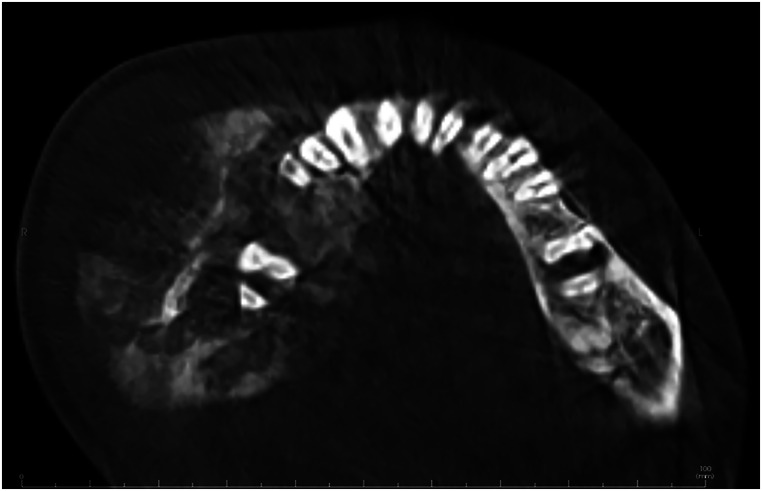
Cone beam computed tomography image shows an intramedullary ill-defined and destructive mass in the right body of the mandible, with extensive periosteal reaction (sunburst appearance) and soft tissue component

On examination, the mandibular mass appeared pushed against the skin, suggesting possible skin involvement. The patient experienced numbness and tenderness upon palpation of the right lower face. Oral examination revealed poor dentition with partial edentulism and no evidence of tooth mobility. A prominent mass was observed on the buccal surface of the right alveolar ridge, extending into the buccal space. No lymphadenopathy was noted on examination. Cone Beam Computed Tomography (CBCT) showed an intramedullary ill-defined and destructive mass in the right body of the mandible, extending approximately from the midline to the ascending ramus. Extensive periosteal reaction with a sunburst appearance was noted (Fig. 1). Based on the radiographic findings, gnathic osteosarcoma and chondrosarcoma were considered.

**Fig. 2:**
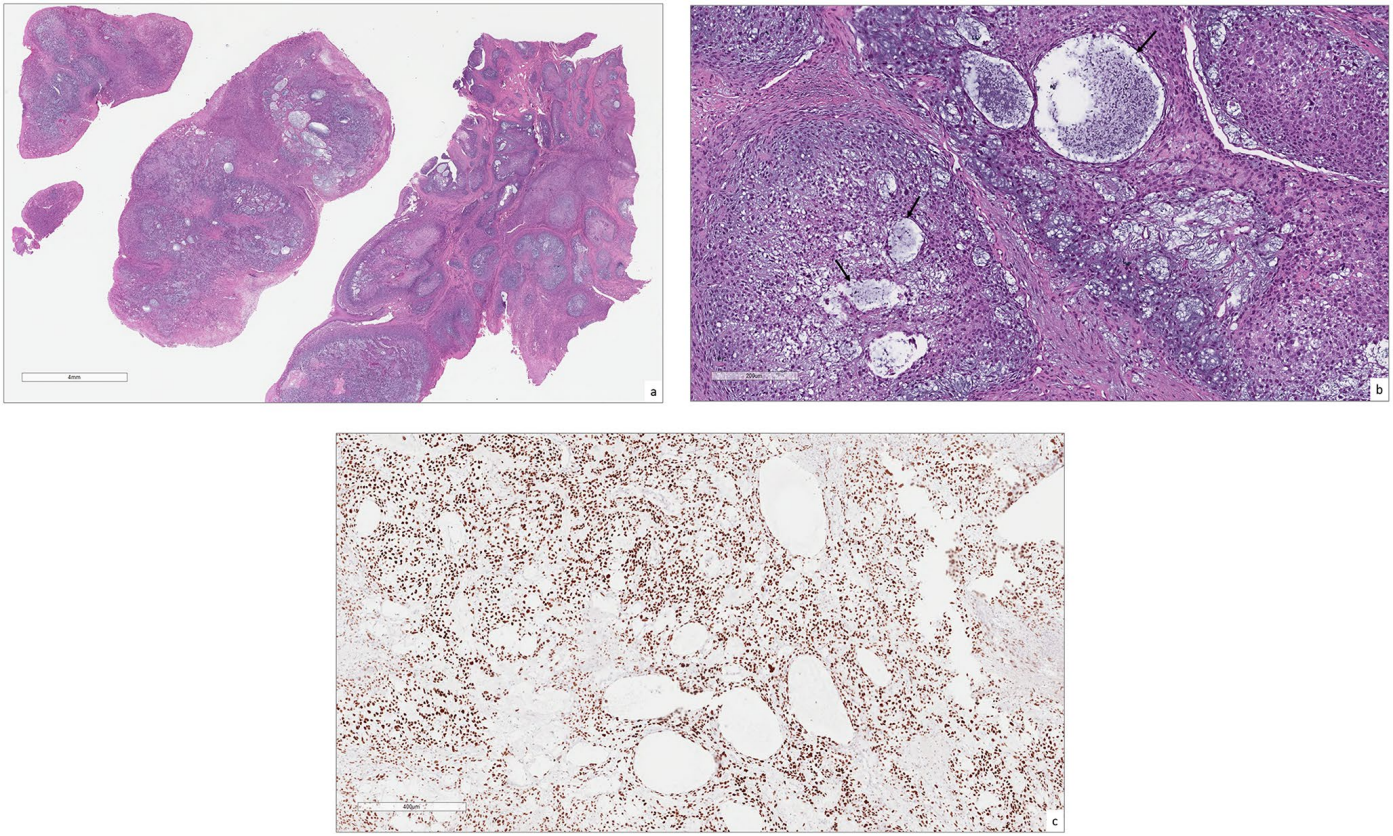
The initial biopsy; **(a)** The tumor is arranged in lobules, displaying a pseudoglandular morphology interspersed with myxoid zones, and covered by ulcerated stratified squamous epithelium, magnification X2 (H&E*). **(b)** Lobules of large, atypical epithelioid and round cells with areas of degenerative cystic spaces, magnification X10 (H&E*). **(c)** Diffuse nuclear expression of SATB2, magnification X4 (IHC**). *Hematoxylin-eosin staining, ** Immunohistochemical staining

An incisional biopsy revealed an ulcerated mass composed of lobules of malignant epithelioid cells forming pseudoglandular and reticular architectures, with degenerative cystic spaces intervening by myxoid zones (Fig. 2a-b). Areas of poorly differentiated, large anaplastic cells with significant nuclear and cellular pleomorphism, as well as numerous atypical mitotic figures are noted. Immunohistochemical (IHC) staining including AE1/AE3, D2-40, S-100, CAM5.2, epithelial membrane antigen (EMA), CD99, GFAP, p40, p63, calponin, and SATB2 were performed. The tumor cells were diffusely positive for SATB2 (Fig. 2c), focally positive for EMA, while the remaining stains were negative. Osteosarcoma was highly suspected; however, due to the lack of osteoid or bone formation and the superficial nature of the initial biopsy, a second, deeper biopsy was recommended. Although SATB2 expression was diffusely positive—a marker with high sensitivity but low specificity for osteoblastic cells [1] —this alone was insufficient for a definitive diagnosis.

**Fig. 3:**
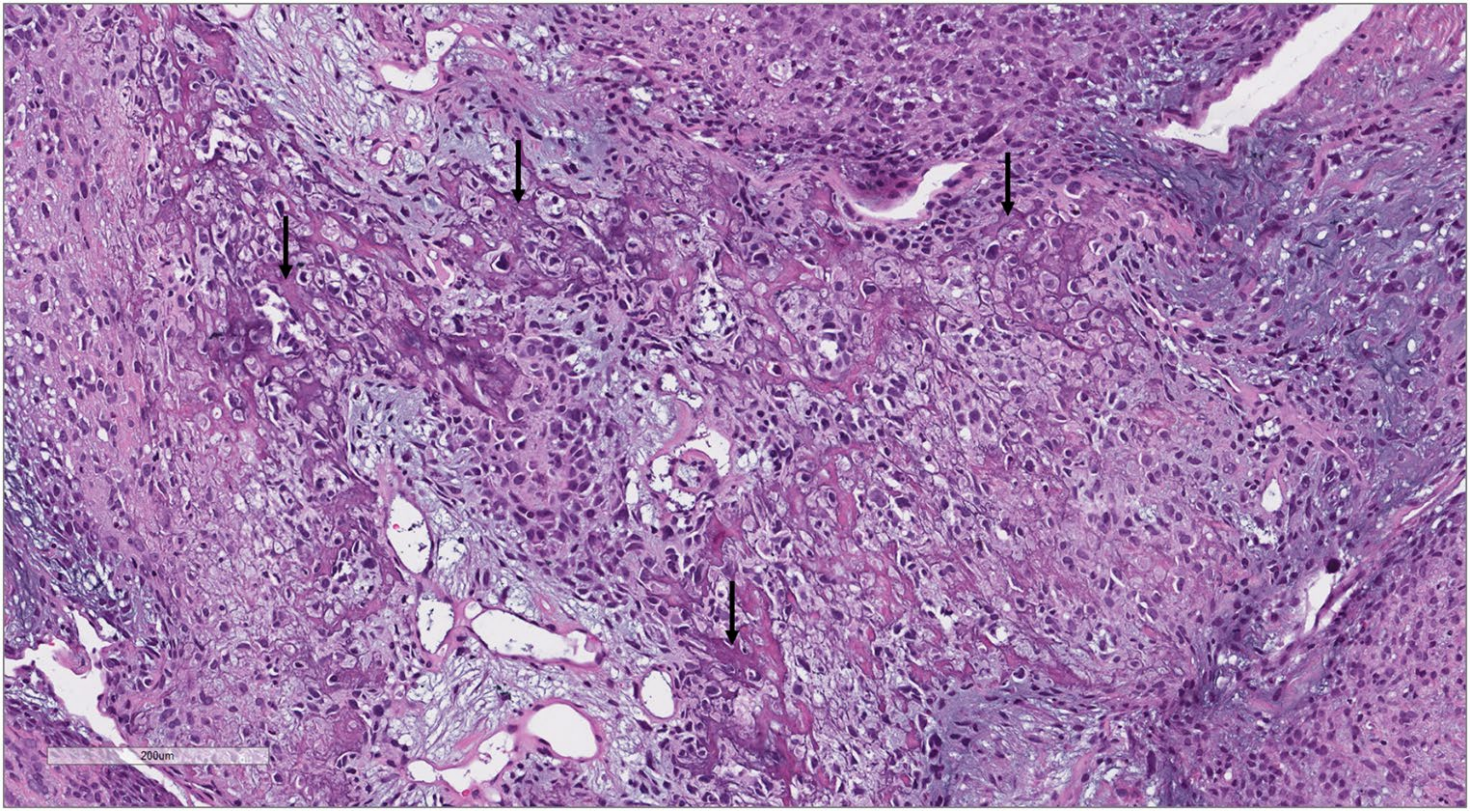
The second biopsy; Osteoid-like wispy calcifications formed by the neoplastic cells, magnification X20 (H&E*). *Hematoxylin-eosin staining

The second biopsy showed similar features noted previously. However, focal areas of osteoid-like wispy calcifications formed by the neoplastic cells were observed, confirming the diagnosis of osteosarcoma (Fig. 3).

The diagnosis of osteosarcoma, particularly in rare presentations such as jaw osteosarcoma, can be challenging due to its varied histopathological features [2, 3]. In this case, the radiographic presentation, along with the presence of osteoid-like wispy calcifications observed in the second biopsy, provided definitive diagnostic clues. However, the initial biopsy did not exhibit osteoid, complicating the diagnosis.

The initial biopsy presented a reticular/pseudoglandular pattern and cystic spaces within myxoid areas, which led to a shift in the diagnostic thought process. These findings could have been mistaken for other malignancies, such as chondrosarcoma. Additionally, the lobulated, glandular architecture and epithelioid morphology raised the possibility of a malignant neoplasm of myoepithelial origin (e.g., myoepithelial carcinoma). This atypical histopathologic presentation necessitated additional ancillary testing and ultimately required a second biopsy to reach a conclusive diagnosis. While the value of IHC staining in the diagnosis of osteosarcoma is limited [1, 4]. SATB2 is commonly used as a marker of osteoblastic differentiation; however, it is not specific for osteosarcoma [5]. Other markers, such as MDM2 and CDK4 are also used in diagnosing osteosarcoma. While they lack specificity, they are particularly useful in distinguishing low-grade osteosarcomas from other benign fibro-osseous lesions [5, 6]. Emerging evidence suggests that Karyopherin α2 (KPNA2) may serve as a novel marker to differentiate osteosarcoma from other bone sarcoma mimics, though its use remains in the early stages of research [7]. This highlights the diagnostic challenges faced, especially when encountered in small biopsy samples.

Radiographic correlation played a crucial role in this case. The sunburst periosteal reaction and the destructive nature of the lesion were instrumental in guiding the diagnostic process toward osteosarcoma. This underscores the importance of integrating clinical, radiographic, and histopathological data to achieve an accurate diagnosis, especially in cases with atypical presentations.

Furthermore, this case emphasizes the need for awareness of the diverse morphological spectrum of osteosarcoma. Pathologists must consider the possibility of unusual presentations and maintain a high index of suspicion when evaluating ambiguous histological features.

## Data Availability

No datasets were generated or analysed during the current study.
